# Supplementary figures and images for: Novel Allosteric Mechanism of Dual p53/MDM2 and p53/MDM4 Inhibition by a Small Molecule
Source: Front Mol Biosci. 2022 Jun 1;9:823195. doi: 10.3389/fmolb.2022.823195 (PMC9198586; doi:10.3389/fmolb.2022.823195)

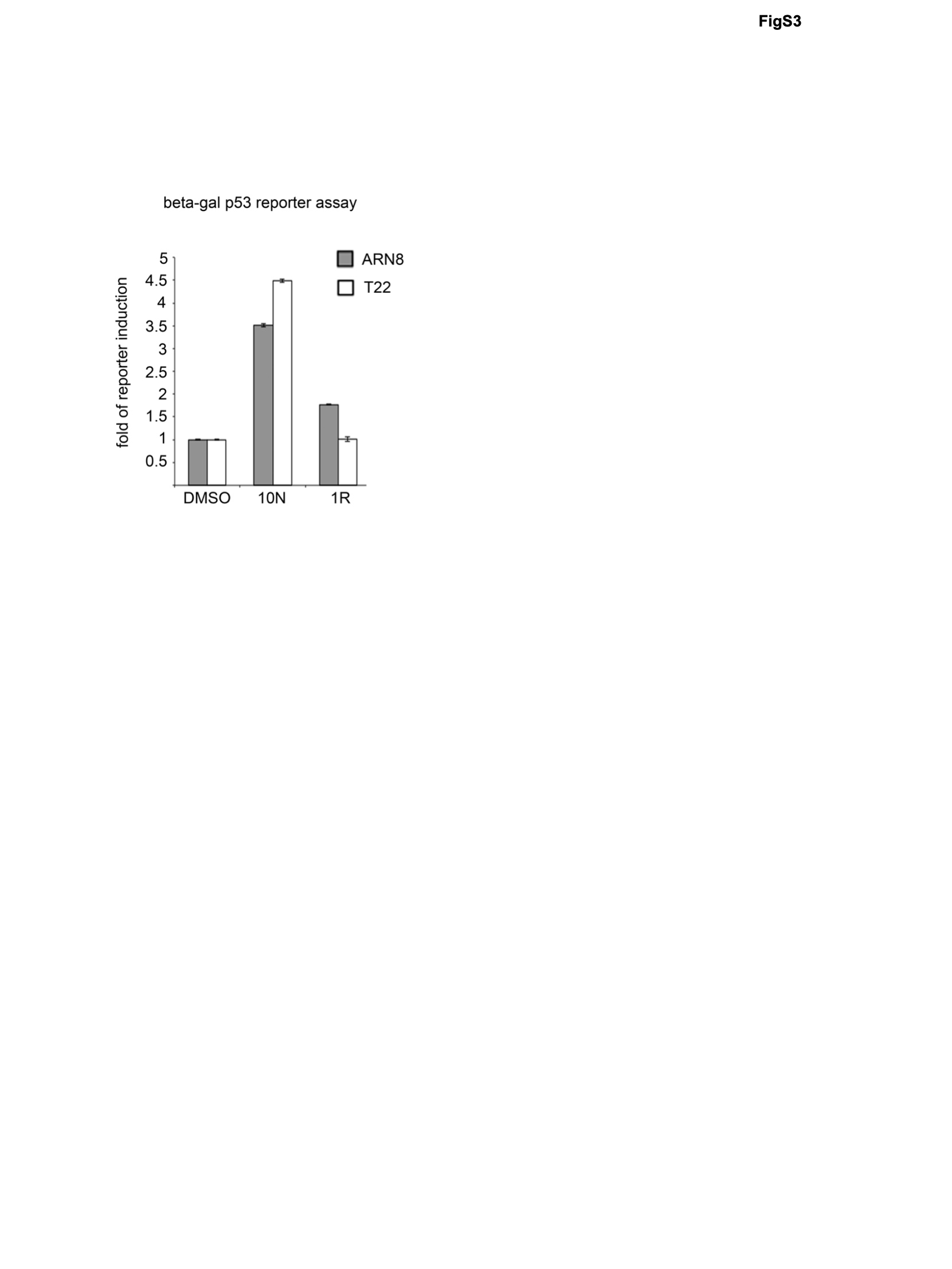

Supplement: Supplementary file 1 [file Image3.TIFF]

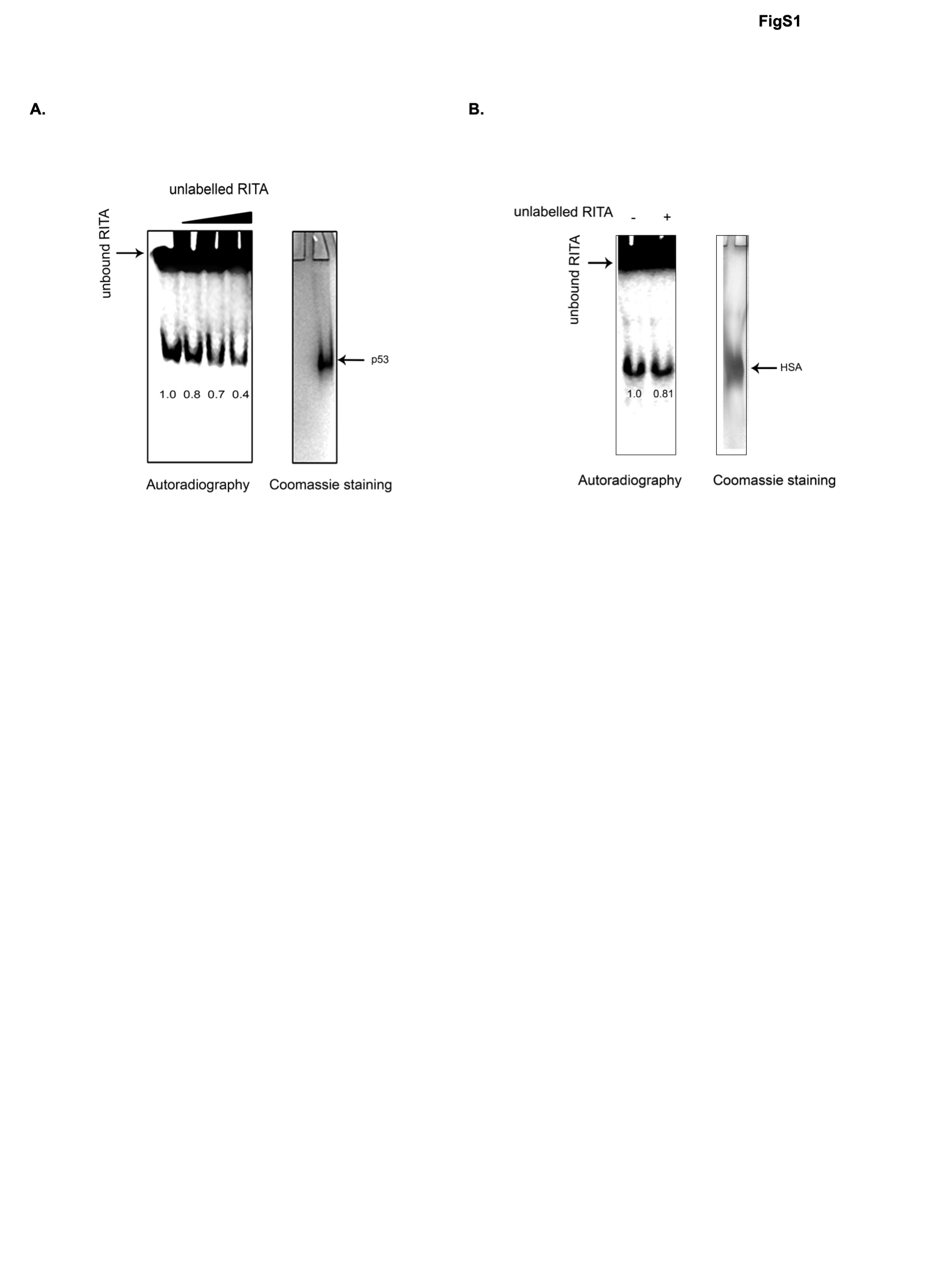

Supplement: Supplementary file 2 [file Image1.TIFF]

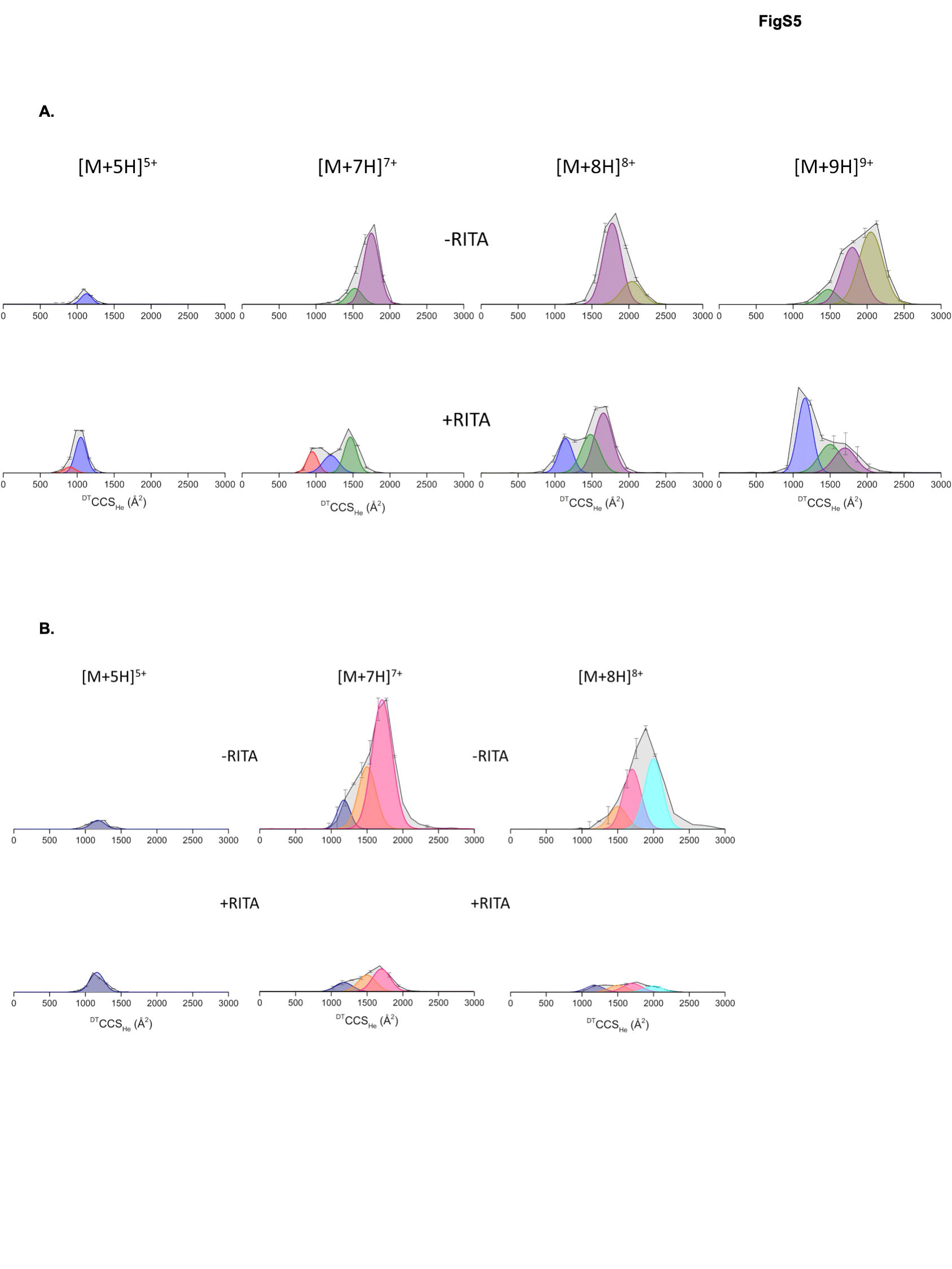

Supplement: Supplementary file 5 [file Image5.TIFF]

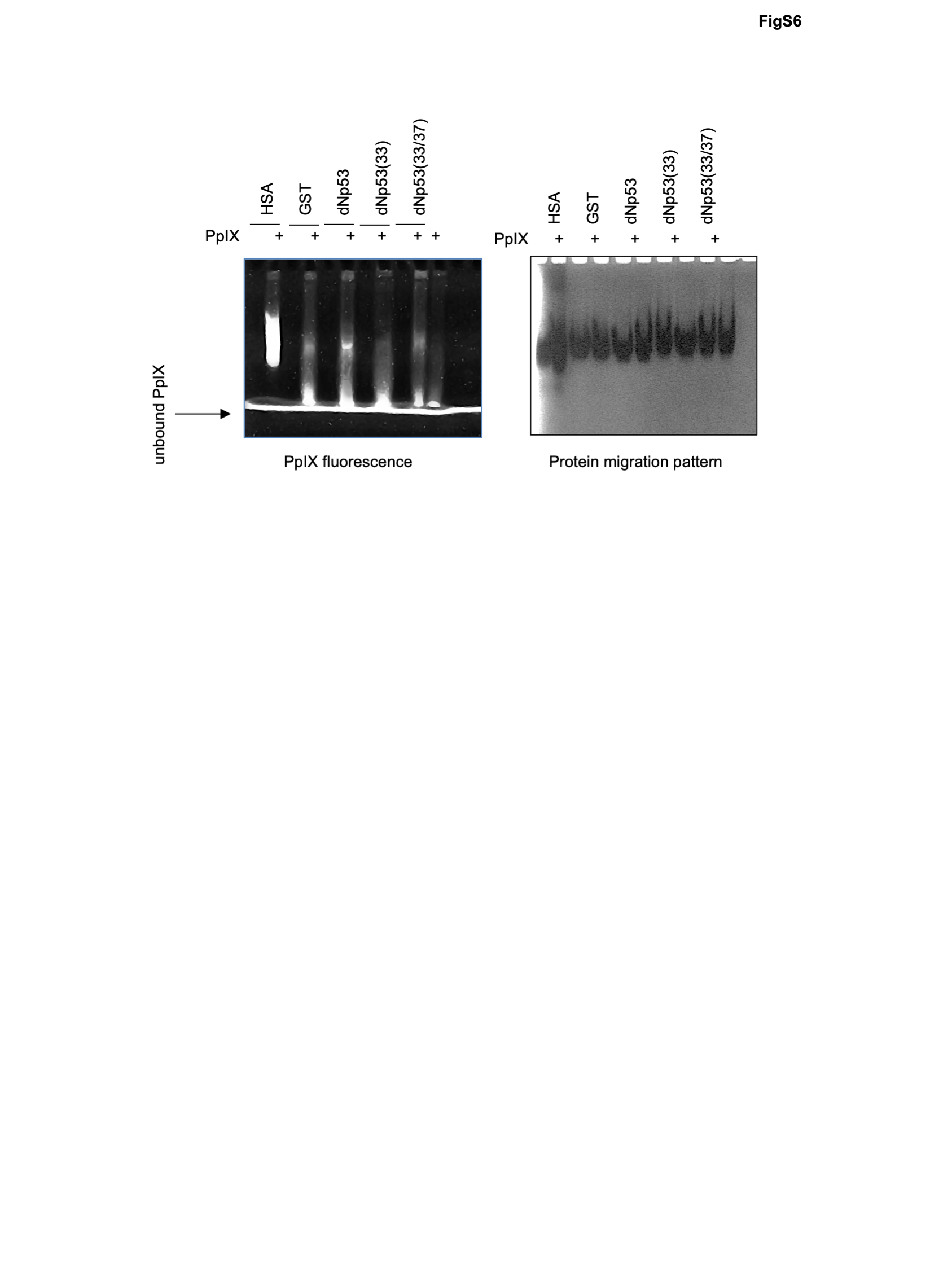

Supplement: Supplementary file 6 [file Image6.TIFF]

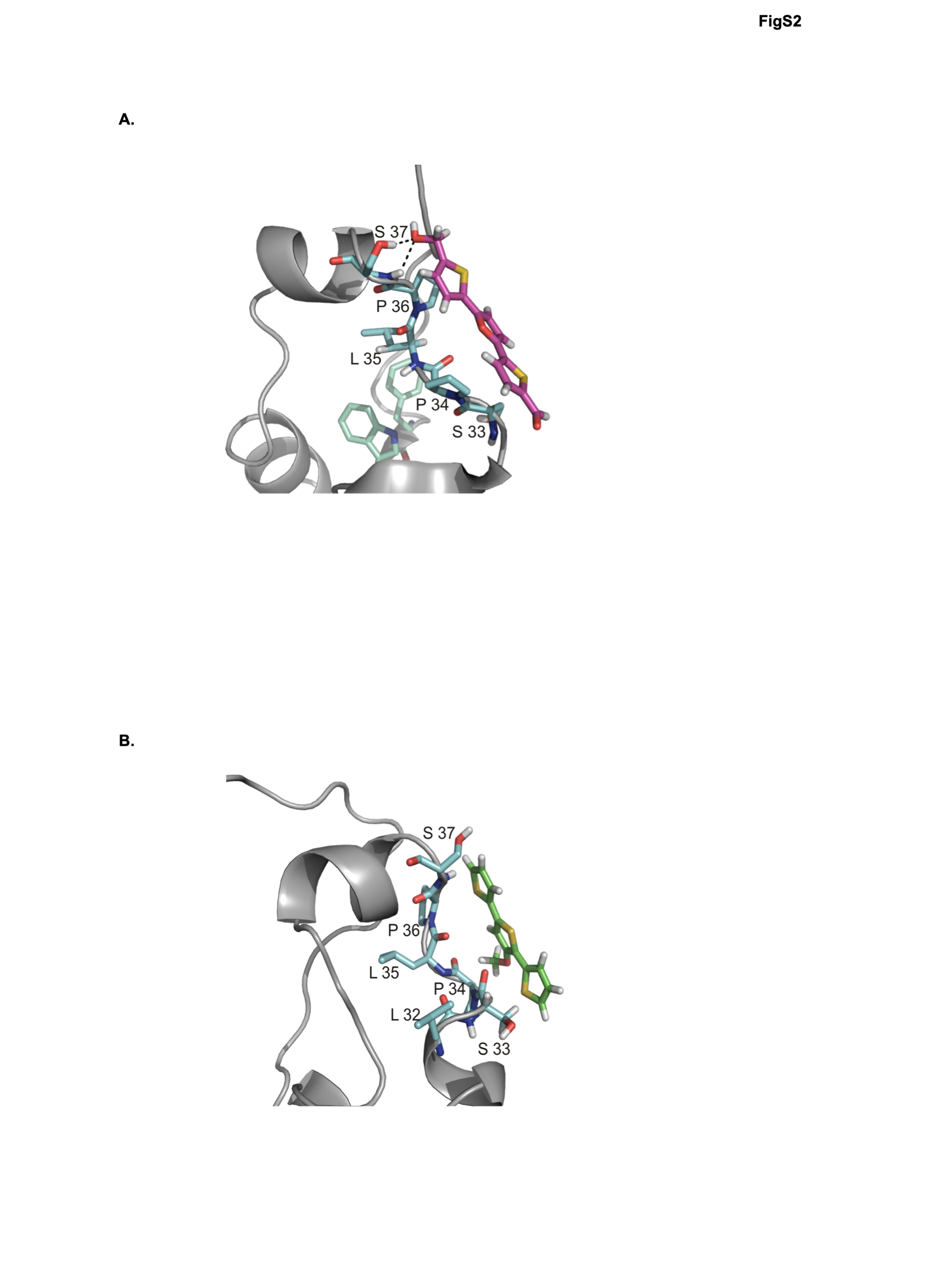

Supplement: Supplementary file 7 [file Image2.TIFF]

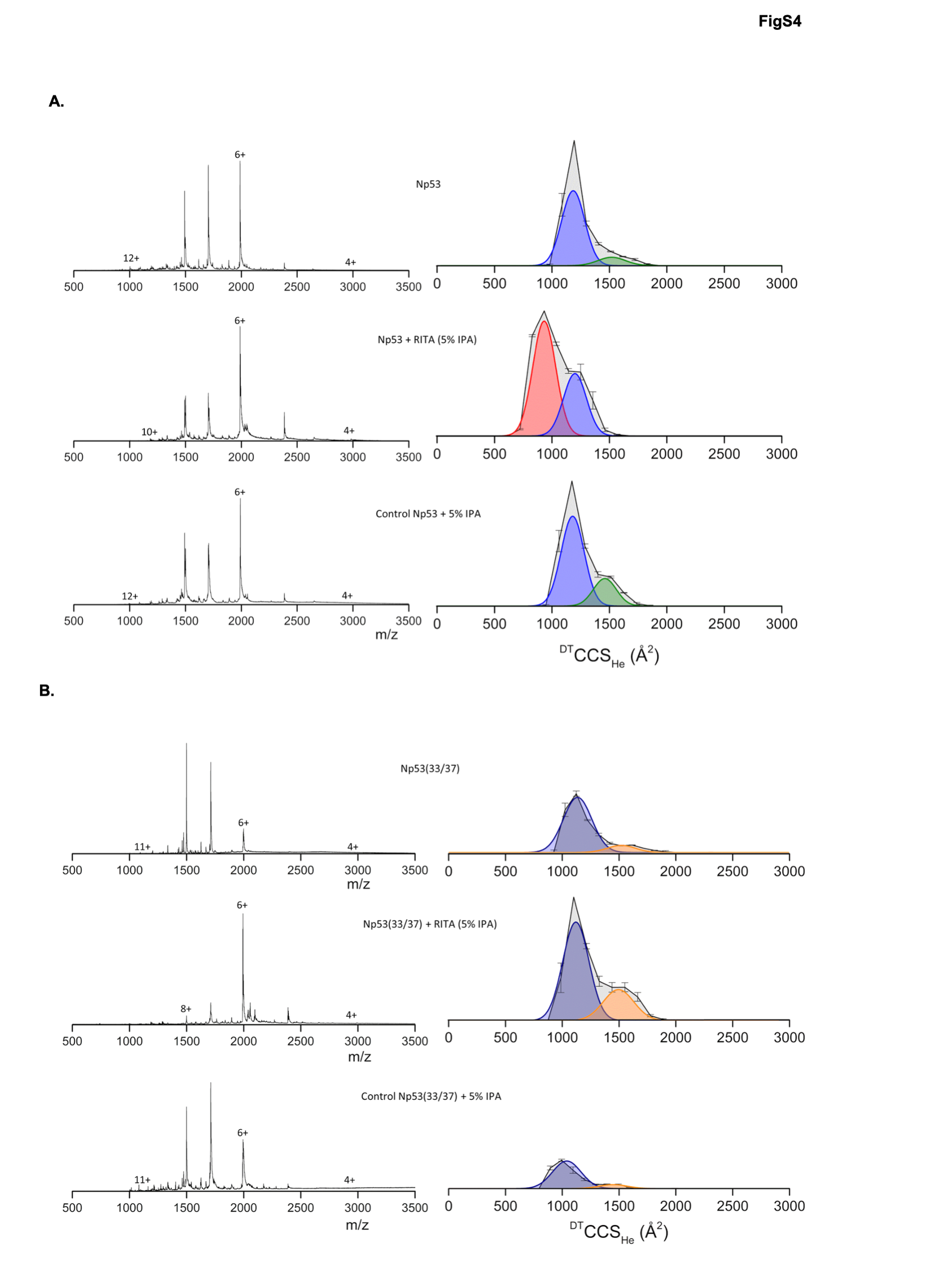

Supplement: Supplementary file 8 [file Image4.TIFF]
