# Supplementary material for: Novel Allosteric Mechanism of Dual p53/MDM2 and p53/MDM4 Inhibition by a Small Molecule
Source: Front Mol Biosci. 2022 Jun 1;9:823195. doi: 10.3389/fmolb.2022.823195 (PMC9198586; doi:10.3389/fmolb.2022.823195)
Supplement: Supplementary file 4 [file Table1.docx]

|  | ID | Structure | Active/inactive |
| --- | --- | --- | --- |
| 1 | RITA |  | Active (RITA)  IC50 ^HCT 116^ 0.26 μM |
| 2 | LCTA-2081 | 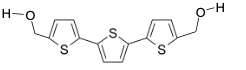 | Active  IC50 ^HCT 116^ 0.38 μM |
| 3 | NSC672170 |  | Active (lower than RITA)  IC50 ^HCT 116^ 0.84 μM |
| 4 | NSC650973 |  | Inactive  IC50 ^HCT 116^ 17.75 μM |
| 5 | NSC629035 |  | Inactive |
| 6 | NSC613590 |  | Inactive |
| 7 | NSC691803 |  | Inactive |
| 8 | NSC647123 |  | Inactive |
| 9 | NSC657767 |  | Inactive |
| 10 | NSC661061 |  | Inactive |
| 11 | NSC694946 |  | Inactive |
| 12 | NSC116644 |  | Inactive |
